# Degradation of iprodione by a novel strain *Azospirillum* sp. A1-3 isolated from Tibet

**DOI:** 10.3389/fmicb.2022.1057030

**Published:** 2023-01-09

**Authors:** Hu Pan, Beike Zhu, Jin Li, Ziqiong Zhou, Wenbin Bu, Yanna Dai, Xiangyang Lu, Huhu Liu, Yun Tian

**Affiliations:** ^1^Institute of Agricultural Product Quality Standard and Testing Research, Tibet Academy of Agricultural and Animal Husbandry Sciences, Lhasa, China; ^2^College of Bioscience and Biotechnology, Hunan Agricultural University, Changsha, China; ^3^Department of Life Sciences, Changzhi University, Changzhi, China; ^4^School of Food Science, Tibet Institute of Agriculture and Animal Husbandry, Nyingchi, China

**Keywords:** *Azospirillum*, novel taxa, iprodione, degradation pathway, bioremediation

## Abstract

A bacterial strain A1-3 with iprodione-degrading capabilities was isolated from the soil for vegetable growing under greenhouses at Lhasa, Tibet. Based on phenotypic, phylogenetic, and genotypic data, strain A1-3 was considered to represent a novel species of genus *Azospirillum*. It was able to use iprodione as the sole source of carbon and energy for growth, 27.96 mg/L (50.80%) iprodione was reduced within 108 h at 25°C. During the degradation of iprodione by *Azospirillum* sp. A1-3, iprodione was firstly degraded to N-(3,5-dichlorophenyl)-2,4-dioxoimidazolidine, and then to (3,5-dichlorophenylurea) acetic acid. However, (3,5-dichlorophenylurea) acetic acid cannot be degraded to 3,5-dichloroaniline by *Azospirillum* sp. A1-3. A *ipaH* gene which has a highly similarity (98.72–99.92%) with other previously reported *ipaH* genes, was presented in *Azospirillum* sp. A1-3. *Azospirillum* novel strain with the ability of iprodione degradation associated with nitrogen fixation has never been reported to date, and *Azospirillum* sp. A1-3 might be a promising candidate for application in the bioremediation of iprodione-contaminated environments.

## Introduction

Iprodione (C_13_H_13_Cl_2_N_3_O_3_, CAS No: 36734-19-7), is a dicarboxamide fungicide that inhibits DNA and RNA synthesis, cell division, and cellular metabolism in fungi (Davidse, 1986), which is commonly used to control fungal infestations by *Botrytis cinerea*, *Alternaria* sp., *Monilinia fructigena*, *Rhizoctonia solani*, *Sclerotinia sclerotiorum*, *Penicillium* sp., *Sclerotinia* sp., and other fungal pathogens in crops (Mukherjee et al., 2003; Morales et al., 2013; Grabke et al., 2014; Campos et al., 2015). Iprodione is moderately persistent in soil, with a half-life of 7–60 days depending on the environmental conditions (Wang et al., 2012; Loutfy et al., 2015), and it has been detected in many samples, such as crops, soil, environmental water, animals, and human urine (Lindh et al., 2007; Grabke et al., 2014; Carneiro et al., 2020; Celeiro et al., 2020). The U.S. environmental protection agency, European commission, and pest management agency of Canada had classified iprodione as a highly toxic to aquatic animals, moderately toxic to plants and birds, and a probable carcinogenic to humans (Verdenelli et al., 2012; Eevers et al., 2017; Bernardes et al., 2019). Thus, the presence of iprodione residues is a matter of serious concern.

Some studies demonstrated that microbial degradation was the primary mechanism for the dissipation of iprodione in the environment (Zhang et al., 2021). To date, several bacterial strains capable of iprodione-degrading have been reported, including *Arthrobacter* sp. MA6, *Pseudomonas* sp., *Arthrobacter* sp. CQH-1, *Microbacterium* sp. YJN-G, *Arthrobacter* sp. C1, *Achromobacter* sp. C2, *Bacillus* sp. KMS-1, and *Paenarthrobacter* sp. YJN-5 (Athiel et al., 1995; Mercadier et al., 1997; Campos et al., 2017; Yang et al., 2017, 2018; Cao et al., 2018; Li, 2018). However, no report has been made on bioremediation of iprodione in Qinghai-Tibet plateau. The objectives of this study were (i) to identify a potential novel taxon (A1-3) with iprodione-degrading capabilities using phenotypic, phylogenetic, and genotypic methods, which was isolated from the soil for vegetable growing under greenhouses at Lhasa, Tibet and (ii) to analyze the degradation characteristic and pathway of iprodione in strain A1-3. It will provide a candidate for the bioremediation of iprodione-contaminated environments.

## Materials and methods

### Chemicals, media, and instruments

Iprodione (purity ≥ 96%), N-(3,5-dichlorophenyl)-2,4-dioxoimidazolidine (purity ≥96%) were purchased from Toronto Research Chemicals Inc.(TRC). N-[[(3,5-dichlorophenyl) amino]carbonyl] glycine (purity ≥ 96%) was synthesized by Shanghai Nafu Biotechnology Co., Ltd. Acetonitrile, acetone, and n-hexane (GC grade) were provided by Fisher Scientific International Inc. Sodium chloride (AR grade) was provided by Chron Chemicals. Luria-Bertani (LB) broth consisted of the following components (g/L): 10.0 tryptone, 5.0 yeast extract, and 10.0 NaCl. Mineral salts medium (MSM) consisted of the following components (g/L): 1.0 NH_4_NO_3_, 1.0 NaCl, 1.5 K_2_HPO_4_, 0.5 KH_2_PO_4_, 0.2 MgSO_4_·7H_2_O, and pH 7.0. Yeast morphology agar (YMA) consisted of the following components (g/L): 14.0 mannitol, 4.5 yeast meal, 0.1 MgSO_4_·7H_2_O, 0.4 K_2_HPO_4_, 0.3 NaCl, 0.01 CaCl_2_, and pH7.0 ± 0.2. Gas chromatograph (6,890 N, ECD with HP-5 Capillary column) was provided by Agilent Technologies. Gas chromatography-tandem mass spectrometer (450GC-320MS, EI with DB-5MS Capillary column) was provided by Bruker. Electronic Balance (JA2003N) was provided by Jinghua instruments. Ultraviolet visible photometer (TU-1901) was provided by Beijing Purkinje General Instrument Co., Ltd. Ultrapure water preparation system (Milli-Q) was provided by Millipore.

### Isolation of iprodione-degrading strain

Iprodione-degrading bacteria were isolated using enrichment culture technique. The samples were collected from the soil for vegetable growing under greenhouses at Lhasa, Tibet (29°66′84.4″N, 90°94′27.6″E, Altitude: 3,667 m). A 5.0 g amount of soil sample was added into a 250 ml flask with 100 ml of sterile MSM containing 100 mg/L iprodione and was incubated on a rotary shaker (180 rpm) at 25°C for 5 days. The suspension (5 ml) was successively transferred to fresh MSM containing 200, 300, and 400 mg/l iprodione and incubated for another 5 days, respectively. After four rounds of enrichment, the culture was diluted and spread onto solid MSM plates containing 100 mg/L iprodione and incubated at 25°C for 7 days. A bacterial named A1-3 with transparent ring was purified for further study.

### Phenotypic characterization and 16S rRNA gene analysis

The phenotypic characteristics of strain A1-3 were tested on yeast mannitol agar (YMA). Cell morphology of strain A1-3 cultured at 25°C for 3 days were observed and photographed by light microscopy (CX31, Olympus). The temperature for optimal growth was tested at 5–40°C (5, 10, 15, 20, 25, 30, 37, and 40°C). The pH range for growth was measured from pH 4.0 to pH 12.0, with an interval of 1.0 units. The salt tolerance was determined with various NaCl concentrations (0, 1, 2, 3, 4, 5, and 6%, w/v). Other biochemical characteristics were carried out according to Ferreira et al. (2020).

Genomic DNA was extracted from strain A1-3 after cultivated in Luria-Broth for 48 h, using MiniBEST Bacterial Genomic DNA Extraction Kit Version 2.0 (TaKaRa Biotechnology Co., Tokyo, Japan). Amplification of 16S rRNA gene was performed under the following conditions: 95°C for 10 min, followed by 94°C for 45 s, 56°C for 45 s, and 72°C for 90 s for 30 cycles with a final 10 min extension at 72°C, the PCR products were detected by agarose gel electrophoresis and then sent to GENEWIZ.lnc for sequencing. Primers used for amplification and sequencing of 16S rRNA was described by Pan et al. (2021). 16S rRNA gene was aligned using EzBioCloud.^1^ Maximum-likelihood (ML) tree was constructed using MEGA7.0 software with bootstrap values of 1,000 replicates (Kumar et al., 2016).

### Genome sequencing and comparative genomic analysis

The genomic DNA of strain A1-3 was sequenced using Illumina and Nanopore platform in MAGIGENE. The genomic sequence information of A1-3 had been submitted to the National Center for Biotechnology Information (NCBI) database under the accession number JAMSLU000000000. Draft genome assemblies were prepared from the ONT reads using Apades v3.11.0, gene prediction using Glimmer 3.02 software. The predicted coding sequences were translated and used as queries to search the COG database.

The digital DNA–DNA hybridization (dDDH) values and confidence intervals were calculated using the recommended settings of Genome-to-Genome Distance Calculator (GGDC; Meier-Kolthoff et al., 2013). The average nucleotide identity (ANI) was determined between strain A1-3 and closely related strains of genus *Azospirillum* by OrthANIu (Yoon et al., 2017). The whole-genome orthologous clusters were compared and analyzed by OrthoVenn2 (Xu et al., 2019). The whole-genome evolution tree were constructed using Type (Strain) Genome Server (Meier-Kolthoff et al., 2022).

### Mensuration of iprodione and the metabolites

Cells of strain A1-3 were cultured in liquid LB medium for 24 h at 25°C and then collected by centrifugation at 8,000 rpm for 5 min. The cell pellets were washed twice with sterilized MSM, adjusted to an optical density at 600 nm (OD_600_) of approximately 1.5, and used as the inoculant. An aliquot of the cells (5%, vol/vol) was inoculated into a 100 ml erlenmeyer flask containing 30 ml of MSM supplemented with 50 mg/L iprodione as the sole source of carbon. The flasks were then incubated at 25°C with shaking (180 rpm). At each sampling point, six flasks were sacrificed for various measurements, three flasks were used to measure the iprodione concentration or for identification of metabolites by GC-ECD or GC–MS/MS, while other three flasks were used to determine the values of OD_600_ of strain A1-3. Each treatment was performed in triplicate, and control experiments (medium without inoculum) were carried out under the same conditions.

Sample preparation of fermentation broth: 20.0 g sample were placed in 150 ml beaker, then 40 ml acetonitrile and 5–6 g NaCl were added, vibration at 180 rpm for 10 min, after 30 min of stratification, 10 ml of supernatant were rotatably evaporated to nearly dry, 5.0 ml acetone with n-hexane (1:9) was used as constant volume for GC-ECD or GC–MS/MS analysis (Celeiro et al., 2020).

The test conditions by Gas chromatography are as follows: HP-5 capillary column (30 m × 0.25 mm × 0.45 μm), carrier gas (N_2_, 99.999% purity), flow rate (3.0 ml/min), flow mode (10:1), sample volume (1 μl), inlet temperature (280°C), heating process: 150°C for 0 min, 15°C/min to 210°C, and 10°C/min to 260°C, 20°C/min to 300°C for 6 min, electron capture detector temperature (230°C).

The test conditions by Gas chromatography-triple tandem quadrupole mass spectrometer are as follows: DB-5MS capillary column (30 m × 0.25 mm × 0.25 μm), carrier gas (N_2_, 99.999% purity), flow rate (1.0 ml/min), no-flow mode, sample volume (1 μl), inlet temperature (230°C), heating process: 60°C for 1 min, 15°C/min to 150°C for 2 min, 10°C/min to 290°C for 4 min. EI mode, electron bombardment energy (70ev), transmission line temperature (280°C), and ion source temperature (230°C). Scan mode was used for qualitative analysis of each component. The scanning quality range was 50–500 amu (Özdoğan et al., 2018; Dai et al., 2022).

### Amplification of *ipaH* and *ddaH* genes

Genomic DNA was extracted from strain A1-3 using MiniBEST Bacterial Genomic DNA Extraction Kit Version 2.0. Amplification of iprodione-degrading genes (*ipaH* and *ddaH*) were performed under the following conditions: 95°C for 5 min, followed by 94°C for 30 s, 56°C for 30 s, and 72°C for 45 s for 32 cycles with a final 10 min extension at 72°C, the PCR products were detected by agarose gel electrophoresis and then sent to GENEWIZ.lnc for sequencing. Primers used for amplification and sequencing of *ipaH* and *ddaH* genes were described by Zhang et al. (2021).

## Results

### Characterization and 16S rRNA gene results of strain A1-3

Colonies of strain A1-3 was white, round, moist, and opaque on YMA solid medium. Strain A1-3 was Gram-strain-negative, curved or slightly curved rods, inmotility, 0.4–0.6 μm × 2.7–3.2 μm. Strain A1-3 grew at 15–30°C and pH 6.0–9.0 (optimum, 20–25°C and pH 7.0–8.0) with 0–2% (w/v) NaCl (optimum, 1%). Negative for oxidase activity, urease activity, and MR-test, while catalase activity, starch hydrolysis, sucrose fermentation, and gelatin hydrolysis were positive.

**Figure 1:**
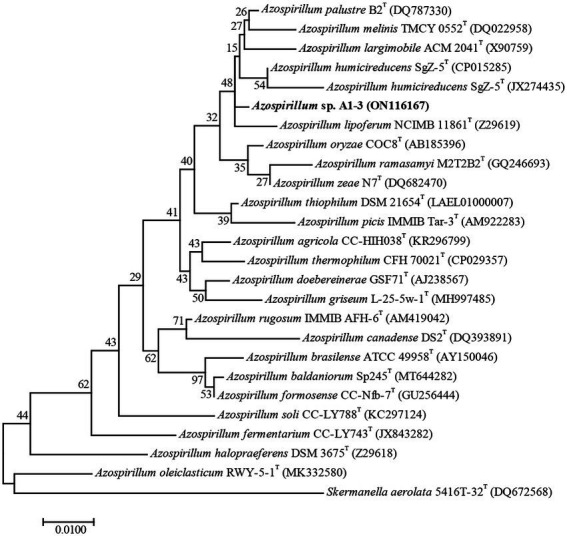
Maximum-likelihood tree based on 16S rRNA gene sequences revealing the relationship between strain A1-3 and other species of the genus *Azospirillum*. *Skermanella aerolata* 5416 T-32^T^ was used as an out-group. Bar, 0.01 substitutions per nucleotide position.

Compared to the sequences deposited in EzBioCloud, the 16S rRNA gene sequence of strain A1-3 was shared the highest similarity with *Azospirillum palustre* B2^T^ (98.85%), followed by *Azospirillum humicireducens* SgZ-5^T^ (98.79%), *Azospirillum oryzae* COC8^T^ (98.65%), *Azospirillum lipoferum* NCIMB 11861^T^ (98.43%), and *Azospirillum melinis* TMCY 0552^T^ (98.35%). A ML tree derived from full 16S rRNA alignments was shown in Figure 1. Phylogenetic analysis of 16S rRNA confirmed its placement within the *Azospirillum* genus, but to form a separate branch of evolution. However, the bootstrap values of the ML tree were low, and the data between the ML tree and the EzBioCloud database were inconsistent, so the taxonomic status of strain A1-3 need to be further confirmed.

### Genomic characteristics and comparative genomics analysis

**Figure 2:**
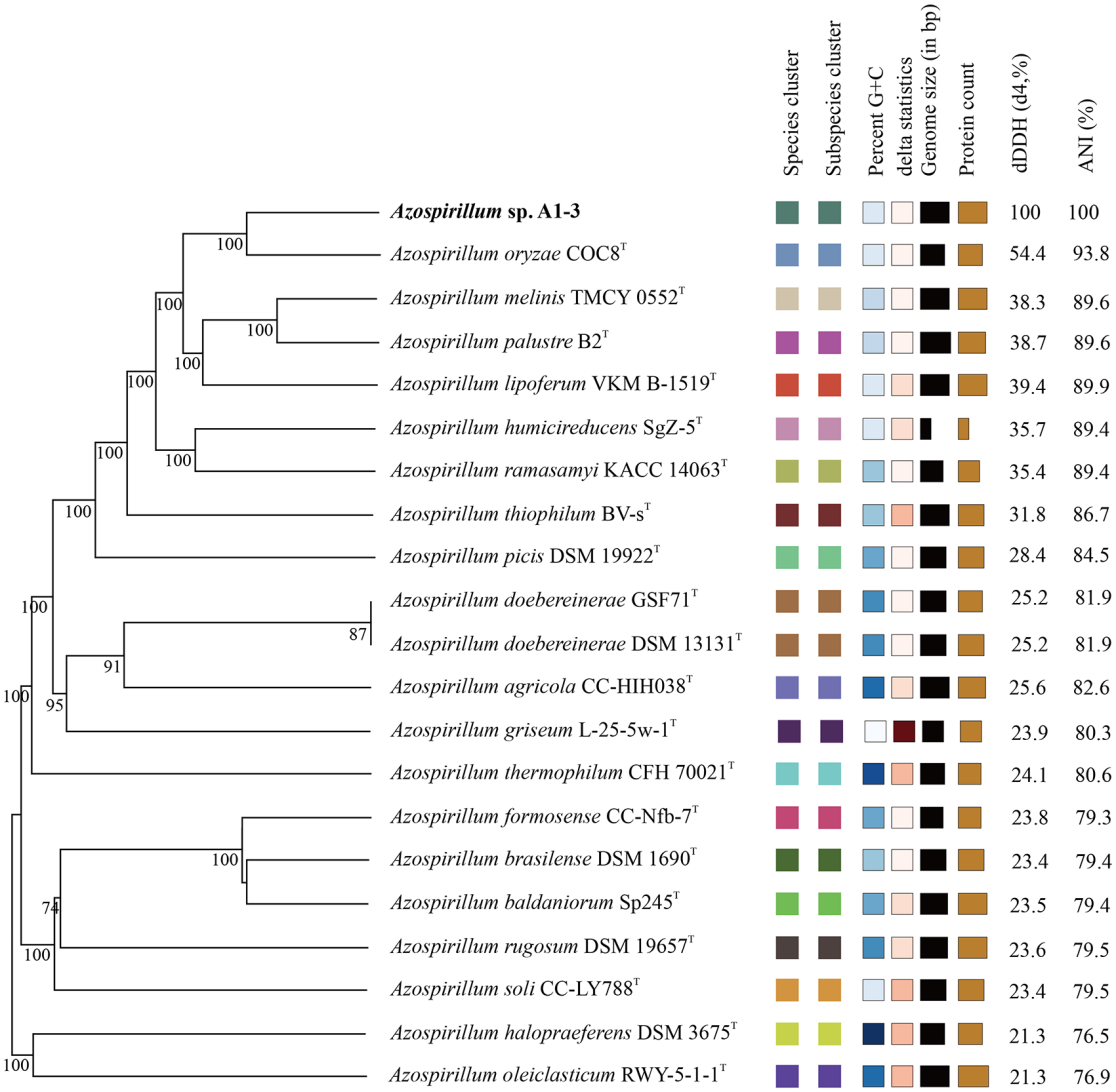
The whole-genome evolution tree of A1-3 and related bacteria with dDDH and ANI values.

**Figure 3:**
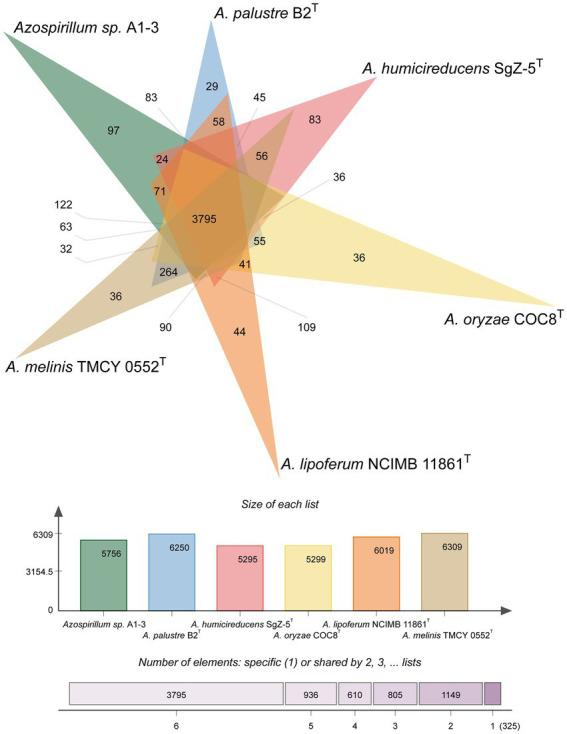
Venn diagram analysis of *Azospirillum* sp. A1-3 and related *Azospirillum* species.

To further confirm the taxonomic status of *Azospirillum* sp. A1-3, the draft genome was sequenced using Illumina and Nanopore platform. The draft genome of *Azospirillum* sp. A1-3 contained nine contigs with an N_50_/N_90_ value of 795,522/606,539 bp. The genome size and DNA G + C content of *Azospirillum* sp. A1-3 were 7.71 Mb and 67.12 mol%, respectively. The whole-genome evolution tree of strain A1-3 and 20 related bacteria shown that strain A1-3 formed a separate lineage with a very high bootstrap support. Furthermore, the dDDH and ANI values between strain A1-3 and other related strains were 21.3–54.4 and 76.5–93.8% (Figure 2), which were lower than the threshold values of 70% and 95–96% for species discrimination (Goris et al., 2007; Meier-Kolthoff et al., 2022). The data from the whole-genome evolution tree were consistent with the EzBioCloud database. So *Azospirillum palustre* B2^T^, *Azospirillum humicireducens* SgZ-5^T^, *Azospirillum oryzae* COC8^T^, *Azospirillum lipoferum* NCIMB 11861^T^, and *Azospirillum melinis* TMCY 0552^T^ were selected for comparative genomic analysis and the genomic properties were listed in Table 1. The orthologous clusters analysis of *Azospirillum* sp. A1-3 and related *Azospirillum* species was shown in Figure 3. *Azospirillum* sp. A1-3, *Azospirillum palustre* B2^T^, *Azospirillum humicireducens* SgZ-5^T^, *Azospirillum oryzae* COC8^T^, *Azospirillum lipoferum* NCIMB 11861^T^, and *Azospirillum melinis* TMCY 0552^T^ were had 5,756, 6,250, 5,295, 5,299, 6,019, and 6,309 proteins, respectively, of which only 3,795 orthologous clusters were identified among all the six strains.

**Table 1:** Genomic properties of *Azospirillum* sp. A1-3 and related strains.

	*Azospirillum* sp. A1-3	*A. palustre* B2^T^	*A. humicireducens* SgZ-5^T^	*A. oryzae* COC8^T^	*A. lipoferum* NCIMB 11861^T^	*A. melinis* TMCY 0552^T^
Assembly accession	GCA_023806445.1	GCF_002573965.1	GCA_001639105.2	GCA_008364795.1	GCA_008364955.1	GCA_017876055.1
Total Size (Mb)	7.71	7.99	6.86	6.75	6.85	7.95
GC (%)	67.1	67.8	67.5	67.4	67.7	67.7
rRNA genes	30	3	14	5	23	4
tRNA genes	92	66	66	63	79	65
Total genes	7,071	7,128	6,054	6,071	6,112	7,111
*nif* symbiotic genes	+	+	+	+	+	+

**Figure 4:**
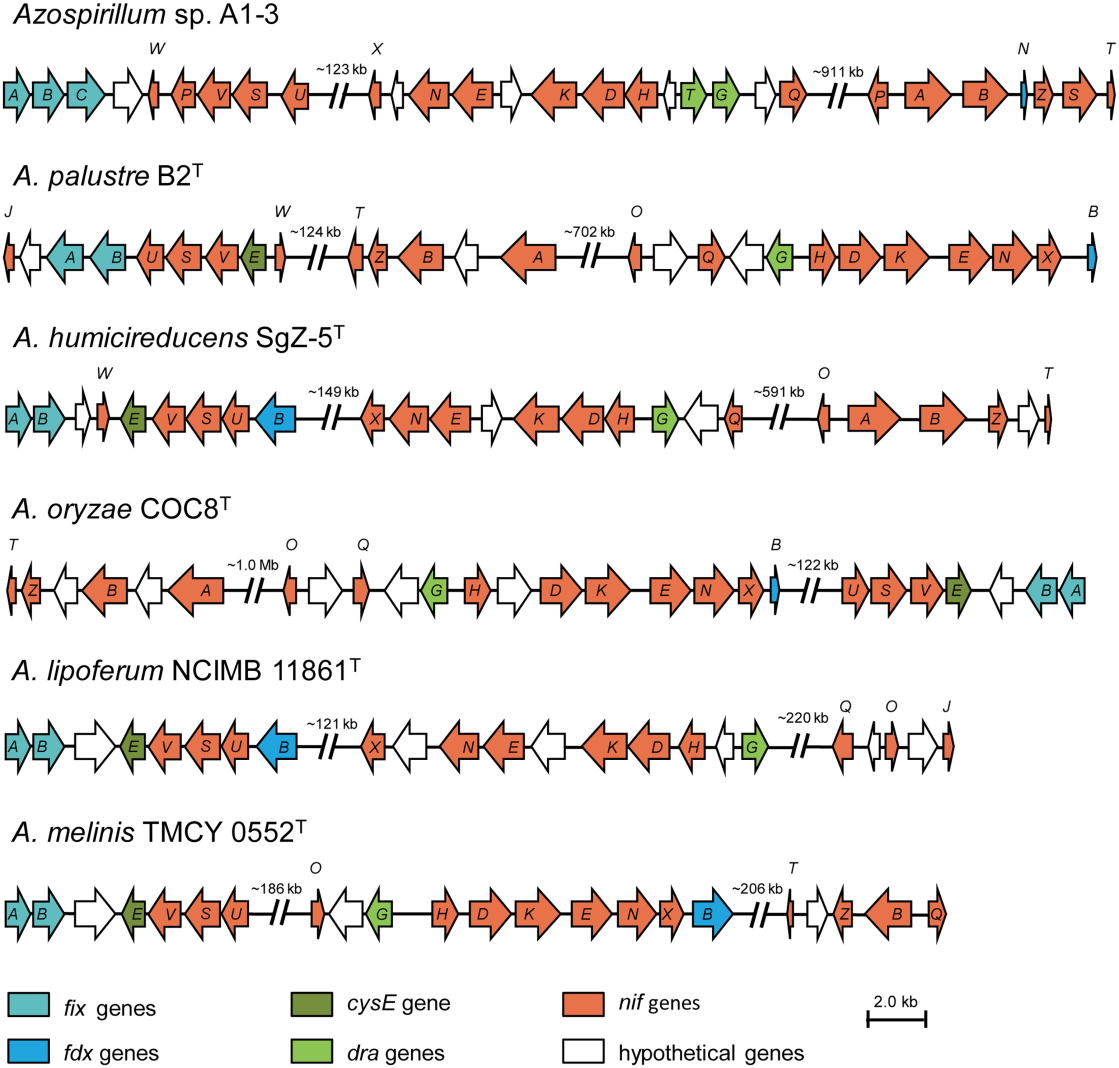
Comparison of the *nif* gene clusters of *Azospirillum* sp. A1-3 with *Azospirillum palustre* B2^T^, *Azospirillum humicireducens* SgZ-5^T^, *Azospirillum oryzae* COC8^T^, *Azospirillum lipoferum* NCIMB 11861^T^, and *Azospirillum melinis T*MCY 0552^T^. The arrow indicates genes transcriptional direction. The hypothetical proteins were colored in white.

A complete set of genes encoding enzymes involved in nitrogen fixation (16,025 bp) was found in the genomic of *Azospirillum* sp. A1-3 (Figure 4). The genetic organization of the *nif* genes performed the high similarity among the genus *Azospirillum*, and were distributed into three portions of the genome. Herein, the first group of genes contained *fixAB* and *nifUSV*. The *fixAB* genes encode a membrane protein complex involved in electron transport to nitrogenase, the *nifUS* genes are generally dedicated to biogenesis of the nitrogenase Fe-S cluster, the *nifV* encode the homocitrate synthase which is an essential component of nitrogenase (Edgren and Nordlund, 2004; Bellés-Sancho et al., 2021; Benoit et al., 2021). The second group contained a series of genes (*nifHDK* and *nifENX*) arranged in the same order. The *nifH* encode the Fe protein and the *nifDK* encode the MoFe protein. NifEN proteins are biosynthetic scaffold for the FeMo-co and NifX is involved in the efficient transfer processes of NifB-co to the NifEN proteins (Fay et al., 2016; Nonaka et al., 2019). The third group of genes contained *nifB* and *nifTZ* involved in synthesis of nitrogenase and *nifA* in *Azospirillum* sp. A1-3*, Azospirillum palustre* B2^T^, *Azospirillum humicireducens* SgZ-5^T^, and Azospirillum oryzae COC8^T^. In addition, *draT* and *draG* genes known to metabolic regulation nitrogenase (Wang et al., 2018) were found in A1-3, but *draT* was not present in *Azospirillum palustre* B2^T^, *Azospirillum humicireducens* SgZ-5^T^, Azospirillum oryzae COC8^T^, *Azospirillum lipoferum* NCIMB 11861^T^, and *Azospirillum melinis* TMCY 0552^T^. All the above analyses confirmed that strain A1-3 was represented a novel species of genus *Azospirillum*.

### Degradation of iprodione by *Azospirillum* sp. A1-3

**Figure 5:**
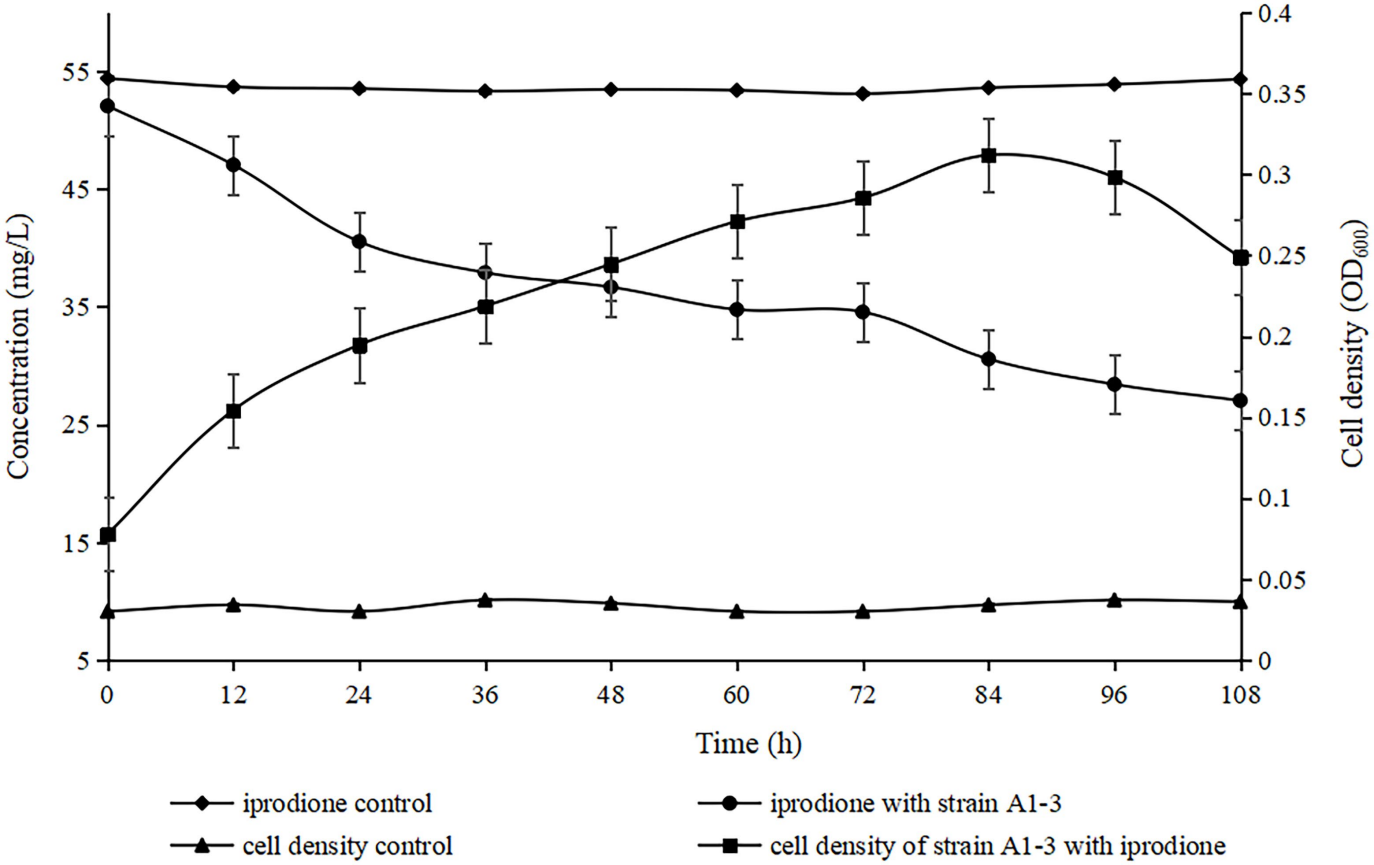
Utilization of iprodione by *Azospirillum* sp. A1-3.

The degradation kinetics of iprodione and growth of *Azospirillum* sp. A1-3 were simultaneously investigated (Figure 5). During the first 84 h, *Azospirillum* sp. A1-3 grew faster, and then there was a slight decrease in conjunction with the decrease of iprodione. After 108 h of incubation, 27.96 mg/L iprodione was reduced by *Azospirillum* sp. A1-3 with the degradation rate of about 50.80%, and the cell density (OD_600_) was increased from 0.078 to 0.249. The results indicated that *Azospirillum* sp. A1-3 could utilize iprodione to support its growth. Herein, it was deduced that strain A1-3 could not completely degrade iprodione but could utilize iprodione as the sole carbon source for its growth.

### Identification of metabolites of iprodione

**Figure 6:**
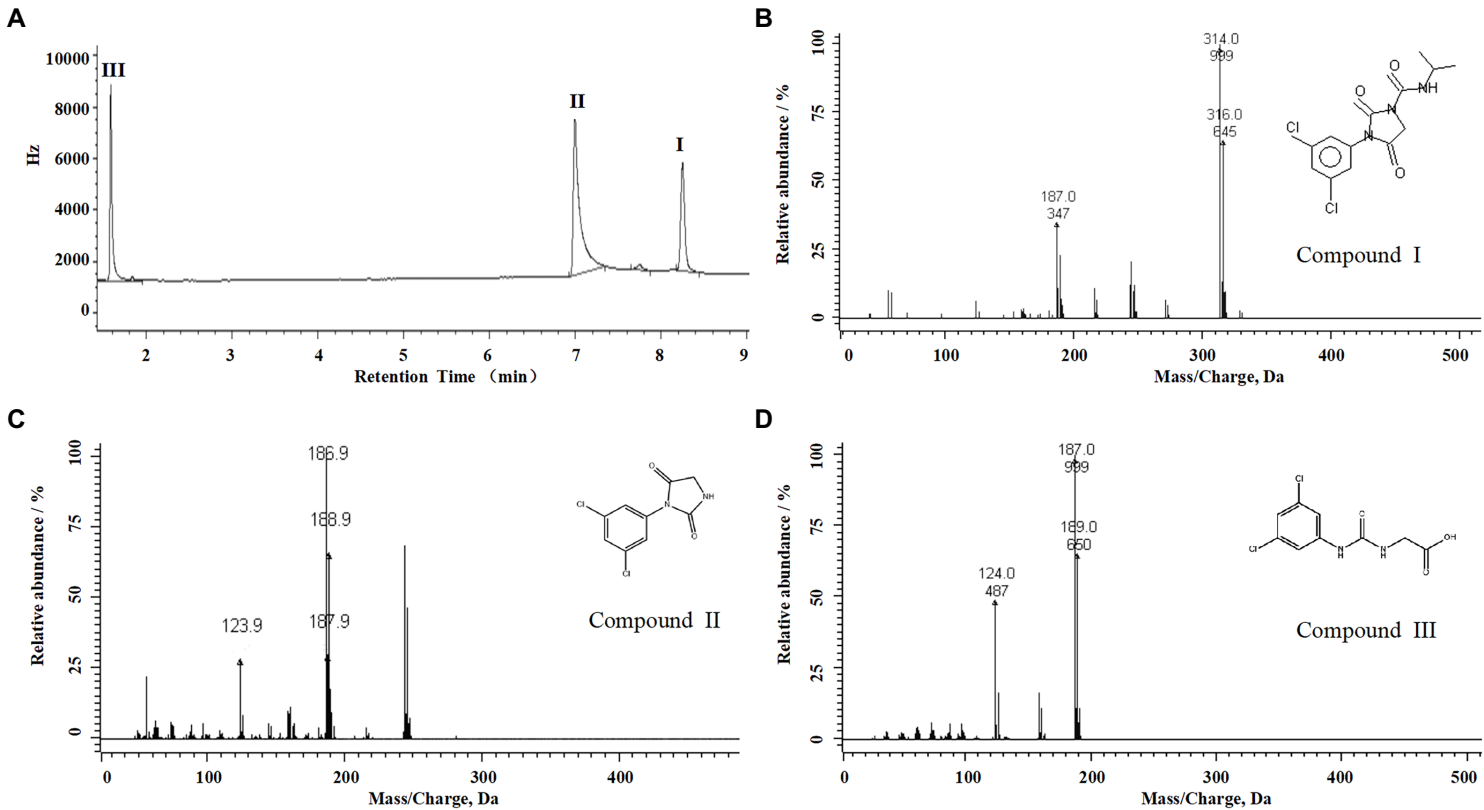
Degradation pathway of iprodione in *Azospirillum* sp. A1-3. **(A)** GC analysis of metabolites that appeared during the degradation of iprodione by strain A1-3, **(B)** MS/MS analysis of compound I, **(C)** MS/MS analysis of compound II, and **(D)** MS/MS analysis of compound III.

**Figure 7:**
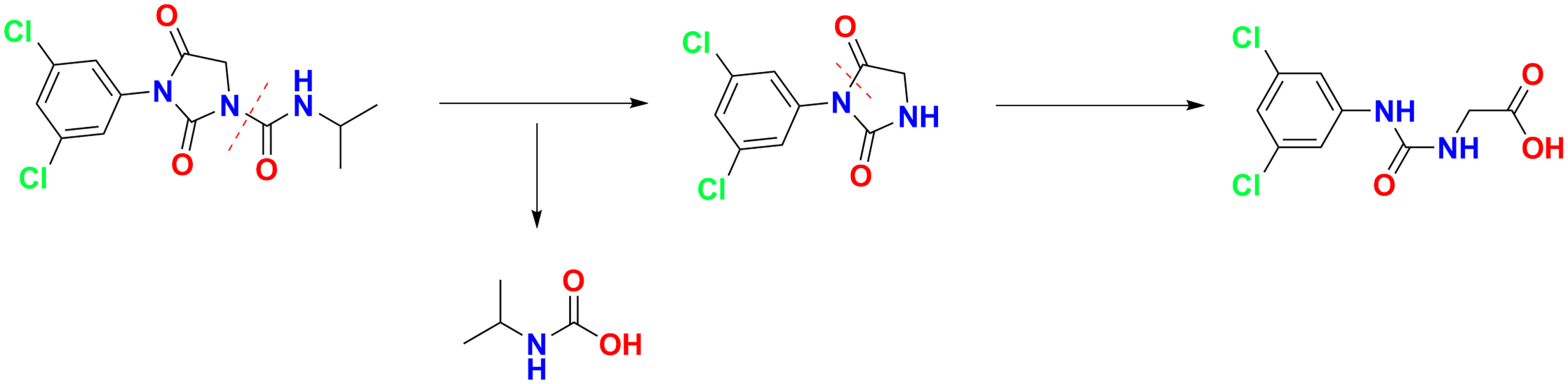
The metabolic pathway of iprodione by *Azospirillum* sp. A1-3.

For the sample collected after inoculation 60 h, three compounds (I, II, and III) were detected at 8.245, 6.990, and 1.598 min by GC-ECD (Figure 6A). The total ion flow diagram of compounds I, II, and III detected by GC-MS/MS were shown in Figures 6B–D, respectively. All compounds contained benzene-ring structures and base peaks of Cl-ion isotopes (Cl ^35-and^ Cl ^37−^). It was found that compound I, II, and III had a prominent peak of Cl-ion isotopes at m/z 314.0999 [C_12_H_10_Cl^35−^_2_N_3_O_3_^+^]/316.645 [C_12_H_10_Cl^37−^_2_N_3_O_3_^+^], 243.9 [C_9_H_6_Cl^35−^_2_N_2_O_2_]/245.9 [C_9_H_6_Cl^37−^_2_N_2_O_2_], and 187.0999 [C_7_H_3_Cl^35−^_2_NO^+^]/189.0660 [C_7_H_3_Cl^37−^_2_NO^+^], respectively. In database of Bruker-NIST, compounds I, II, and III were identified as iprodione, N-(3,5-dichlorophenyl)-2,4-dioxoimidazolidine and (3,5-dichlorophenylurea) acetic acid, which was the same as the typical one (Yang et al., 2018). However, (3,5-dichlorophenylurea) acetic acid could not be degraded to 3,5-dichloroaniline by *Azospirillum* sp. A1-3, this may be related to the deletion of related genes (Zhang et al., 2021). The metabolic pathway of iprodione by *Azospirillum* sp. A1-3 was shown in Figure 7.

### The amplification results of *ipaH* and *ddaH* genes

**Figure 8:**
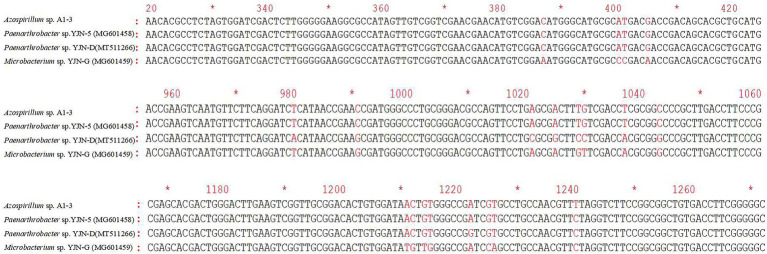
The differences of *ipaH* gene sequences in *Azospirillum* sp. A1-3 and other iprodione-degrading strains.

The *ipaH* gene was responsible for hydrolyzing the N1 amide bond of iprodione, and the *ddaH* gene was responsible for hydantoin ring cleavage of N-(3,-5-dichlorophenyl)-2,4-dioxoimidazolidine (Zhang et al., 2021). The PCR amplification results of *ipaH* and *ddaH* genes in *Azospirillum* sp. A1-3 were shown that the *ipaH* gene was a distinct single band, while the *ddaH* gene was diffuse and could not be sequenced. The sequencing result of *ipaH* gene was shown that it has a highly similarity (98.72–99.92%) with other reported *ipaH* genes (Figure 8). While the previously reported *ddaH* gene were not presented in *Azospirillum* sp. A1-3, other types of hydrolases maybe involved in the process of hydantoin ring cleavage of N-(3,-5-dichlorophenyl)-2,4-dioxoimidazolidine in *Azospirillum* sp. A1-3.

## Discussion

Iprodione was a very popular fungicide used in many kinds of crops all over the world, with microbial degradation being the main way to its environmental dissipation. Several bacterial strains capable of iprodione-degrading have been reported, but not *Azospirillum* spp. Herein a novel *Azospirillum* sp.A1-3 with iprodione-degrading capabilities was reported. *Azospirillum* novel strains with the ability of iprodione degradation associated with nitrogen fixation has never been reported to date. *Azospirillum* was contained 24 validly published species and nine not validly published species^2^ at the time of writing. Some studies have shown that *Azospirillum* spp. not only have nitrogen-fixing function, but also have some other functions, such as heavy oil degrading, atrazine degrading, denitrification ability, carotenoids produce (Jang et al., 2019; Liu et al., 2019; Wu et al., 2020; Mishra et al., 2021), and the functional diversity of *Azospirillum* spp. needs to be studied future.

Previous studies have shown that the initial concentration, degradation rate, and time of iprodione in different microorganisms were 1.5 mM/L–100 mg/L, 41.4–100% and 20 h-10 days, respectively (Table 2; Athiel et al., 1995; Mercadier et al., 1997; Campos et al., 2017; Yang et al., 2017, 2018; Cao et al., 2018; Li, 2018). In this study, 50.80% iprodione was degraded by *Azospirillum* sp. A1-3 after 108 h, and iprodione could been firstly degraded to N-(3,5-dichlorophenyl)-2,4-dioxoimidazolidine, and then to (3,5-dichlorophenylurea) acetic acid. The *Pseudomonas* spp. and *Microbacterium* spp. could quickly degraded iprodione within 24 h, while the degradation pathway and molecular mechanism of iprodione in these strains were not resolved. The degradation pathway of iprodione in *Azospirillum* sp. A1-3 was partly the same as the typical one in *Paenarthrobacter* sp. YJN-5 &YJN-D, while the initial tolerance concentration of iprodione of strain A1-3 were higher than them. Some studies demonstrated that the coding genes involved in the above-mentioned processes had a highly similarity (Zhang et al., 2021). In our follow-up study, the *ipaH* gene, which was responsible for the initial step in the iprodione degradation pathway, have a 98–99% similarity in many kinds of microorganisms (*Acinetobacter* sp., *Paenarthrobacter* sp., *Microbacterium* sp., and *Azospirillum* sp., part of the data does not show; Zhou, 2022). The difference copy numbers or mutation of amino acid site of iprodione-degrading genes maybe are the mainly reasons for the different degradation rate of iprodione in microorganisms. The molecular mechanism of different degradation rate of iprodione with the highly similarity iprodione-degrading genes (*ipaH*, *ddaH*, and *duaH*) in different genera of microorganisms have need to be further studied.

**Table 2:** Basic characteristics of iprodione-degrading strains.

Source	Species of isolates	Initial concentration of iprodione	Degradation rate	Degradation time	Degradation genes
Soil	*Arthrobacter* sp. MA6	9.90 mg/L	86.7%	7 days	ND
Soil	*Pseudomonas fluorescens, Pseudomonas* sp.*, P. paucimobilis*	8.25 mg/L	100%	20–24 h	ND
Soil	*Zygosaccharomyces rouxii* DBVPG 6399	1 mg/L	100%	9 days	ND
Farmland soil	*Arthrobacter* sp.CQH	100 mg/L	100%	112 h	ND
Soil	*Microbacterium* sp. CHQ-1	100 mg/L	100%	96 h	ND
Activated Sludge	*Microbacterium* sp.YJN-G	100 mg/L	100%	24 h	*ipaH*
Acidic soil	*Arthrobacter* sp. C1, *Achromobacter* sp. C2	60 mM/L	100%	10 days	ND
Soil	*Bacillus* sp.KMS-1	25 mg/L	41.4%	7 days	ND
Grapes grow soil	*Paenarthrobacter* sp. YJN-5 *Paenarthrobacter* sp. YJN-D	1.5 mM/L	95%	80 h	*ipaH, ddaH, and duaH*

## Data availability statement

The datasets presented in this study can be found in online repositories. The names of the repository/repositories and accession number(s) can be found in the article/supplementary material.

## Author contributions

HL and YT initiated and designed the research. HP and BZ performed the experiments. HP, JL, ZZ, WB, YD, and XL analyzed the data. BZ, JL, and HP prepared materials. HP drafted the manuscript. All authors contributed to the article and approved the submitted version.

## Funding

This work was supported by the National Natural Science Foundation of China (32060025), the Science and Technology Innovation Program of Hunan Province (2022RC1170), the Training Program for Excellent Young Innovators of Changsha (kq2106049), the Provincial College Students Innovative Entrepreneurial Training Plan Program of Hunan, China (S202110537006X), College students’ Innovative Entreneurial Training Plan Program of Hunan Agricultural University, China (S202010537078), and the Double first-class construction project of Hunan Agricultural University (SYL201802002).

## Conflict of interest

The authors declare that the research was conducted in the absence of any commercial or financial relationships that could be construed as a potential conflict of interest.

## Publisher’s note

All claims expressed in this article are solely those of the authors and do not necessarily represent those of their affiliated organizations, or those of the publisher, the editors and the reviewers. Any product that may be evaluated in this article, or claim that may be made by its manufacturer, is not guaranteed or endorsed by the publisher.

## Footnotes

^1^ https://eztaxon-e.ezbiocloud.net/

^2^ https://lpsn.dsmz.de/Azospirillum
